# A Regional Smoothing Block Sparse Bayesian Learning Method With Temporal Correlation for Channel Selection in P300 Speller

**DOI:** 10.3389/fnhum.2022.875851

**Published:** 2022-06-10

**Authors:** Xueqing Zhao, Jing Jin, Ren Xu, Shurui Li, Hao Sun, Xingyu Wang, Andrzej Cichocki

**Affiliations:** ^1^The Key Laboratory of Smart Manufacturing in Energy Chemical Process, Ministry of Education, East China University of Science and Technology, Shanghai, China; ^2^Shenzhen Research Institute of East China University of Technology, Shenzhen, China; ^3^g.tec medical engineering GmbH, Graz, Austria; ^4^Skolkovo Institute of Science and Technology, Moscow, Russia; ^5^Systems Research Institute of Polish Academy of Science, Warsaw, Poland; ^6^Department of Informatics, Nicolaus Copernicus University, Toruń, Poland

**Keywords:** channel selection, sparse bayesian learning, temporal correlation, brain-computer interface, EEG, P300

## Abstract

The P300-based brain–computer interfaces (BCIs) enable participants to communicate by decoding the electroencephalography (EEG) signal. Different regions of the brain correspond to various mental activities. Therefore, removing weak task-relevant and noisy channels through channel selection is necessary when decoding a specific type of activity from EEG. It can improve the recognition accuracy and reduce the training time of the subsequent models. This study proposes a novel block sparse Bayesian-based channel selection method for the P300 speller. In this method, we introduce block sparse Bayesian learning (BSBL) into the channel selection of P300 BCI for the first time and propose a regional smoothing BSBL (RSBSBL) by combining the spatial distribution properties of EEG. The RSBSBL can determine the number of channels adaptively. To ensure practicality, we design an automatic selection iteration strategy model to reduce the time cost caused by the inverse operation of the large-size matrix. We verified the proposed method on two public P300 datasets and on our collected datasets. The experimental results show that the proposed method can remove the inferior channels and work with the classifier to obtain high-classification accuracy. Hence, RSBSBL has tremendous potential for channel selection in P300 tasks.

## Introduction

Brain–computer interface (BCI) is a direct interactive pathway designed to establish a non-muscle connection between the human brain and the computer (Wolpaw et al., 2002; Jin et al., 2015). It provides a new way to communicate with the outside, for example, daily communication (Sorbello et al., 2017; He et al., 2019) and wheelchair control (Kim et al., 2016; Deng et al., 2019). In addition, BCIs can also be used to aid in the diagnosis of disorders of consciousness (Maestú et al., 2019; Ando et al., 2021). BCIs can be divided into invasive and non-invasive ones. Electroencephalography (EEG) is a non-invasive technique that records brain signals through electrodes placed on the scalp. Generally, users’ brain signals are recorded, amplified, and pre-processed with an EEG recorder, and then the signals are converted to commands *via* classifiers (Bashashati et al., 2007). Currently, BCIs based on the Event-Related Potential (ERP) (Hoffmann et al., 2008a; Lopez-Calderon and Luck, 2014), Steady-State Visual Evoked Potential (SSVEP) (Nakanishi et al., 2017), and Motor Imagery (MI) (Padfield et al., 2019) are the three main research directions. The oddball paradigm is a typical paradigm of P300, where standard and deviant stimuli are included. These two kinds of stimuli appear randomly with large and small probabilities, and deviant stimuli are the targets in small probability events that correspond to the spelling character (Donchin et al., 2000). The spelling paradigms and algorithms based on P300 have been widely developed in recent years (Cecotti and Graser, 2010; Townsend et al., 2010; Hammer et al., 2018; Arvaneh et al., 2019; Jin et al., 2019; Huang et al., 2022). This study is focused on the P300 BCI system.

To provide a complete coverage of regions related to EEG activity, a large number of electrodes are used for EEG acquisition. An electrode is regarded as a channel. However, a realistic EEG system typically uses the data of a small number of channels during computation to minimize the preparation time and cost (Cecotti et al., 2011). Channel selection helps to exclude the weak task-relevant and noisy channels, thus improving the classification accuracy and reducing the classifier training time. Inter-participant differences and equipment differences can make the best subset of channels in the same paradigm different. The flexibility of selecting a subset of empirical channels in the complex BCI data is insufficient, and the data-based channel selection method is more conducive to giving the optimal channel selection. Therefore, the method of automatically determining a subset of channels has better application prospects than selecting a fixed subset.

Different evaluation approaches, such as filter, wrapper, embedded, hybrid, and human-based techniques have been widely used to select features and the subset of channels in the P300 speller (Alotaiby et al., 2015). Filters like Fisher Score (Lal et al., 2004) are usually independent of the classifier and select channels based on the relevance. A CCA spatial filter also proved to be effective in event-related signal processing (Reichert et al., 2017). On the other hand, wrappers select the channel set according to the algorithm effect and search for channels through continuous heuristic methods. Support Vector Machine based recursive channel elimination (SVM-RCE) can be considered a typical example of a wrapper (Rakotomamonjy and Guigue, 2008). The hybrid approach is a combination of filter and wrapper and uses the wrapper to obtain a subset of the available channels after handling the filter (Liu and Yu, 2005). The human-based approaches are the methods in which the experienced experts select channels by analyzing certain technical indicators (Tekgul et al., 2005). In addition, some channel selection algorithms are based on evolutionary algorithms, such as Particle Swarm Optimization (PSO), which also belong to wrappers (Martinez-Cagigal and Hornero, 2017; Arican and Polat, 2019). For embedded methods, the selection is usually implicit and integrated with the learner training process. By giving sparse weight to features or channels, sparse methods can obtain a classifier that needs fewer selected features or channels. The Least Absolute Shrinkage and Selectionator operator (LASSO), a linear regressor with *L_1_* regularization, can be regarded as an embedded method (Tibshirani, 1996). In EEG research, LASSO has also become a commonly used feature selection algorithm and extended to channel selection (Tomioka and Müller, 2010). Yuan extended the LASSO method to groups in 2006, giving birth to the group LASSO (GLASSO), which allows us to group all variables and then penalize the *L_2_* parametrization of each group in the objective function, thus achieving the effect of eliminating a whole group of coefficients to zero at the same time (Yuan and Lin, 2006). The Bayesian framework-based feature selection and classification methods are widely used in EEG. Studies have shown the outstanding performance of Bayesian linear discriminant analysis (BLDA) in EEG decoding (Hoffmann et al., 2008a; Lei et al., 2009; Manyakov et al., 2011). Tipping et al. proposed a sparse Bayesian learning (SBL) method under the Bayesian framework to solve the regression problem (Tipping, 2001). SBL can complete the feature selection of P300 through sparsity (Hoffmann et al., 2008b) and has been used for channel selection (Wu et al., 2014; Zhang et al., 2017; Dey et al., 2020). EEG is a common response of regional neurons (Hassan and Wendling, 2018). However, the channel optimization approach described above does not consider the spatial structure between the channels of EEG signals. In addition, a few existing algorithms consider the temporal correlation in a single channel, which means the amplitude correlation between time points within each channel.

This paper proposes a regional smoothing SBL (RSBSBL) method for channel selection of the P300 signal. Block sparse Bayesian learning (BSBL) was first proposed for sparse signal recovery (Zhang and Rao, 2011). It is the first time that the BSBL is applied to EEG channel selection. The P300 features are usually filtered and down-sampled in the temporal series, and features from the same channel are correlated. In this method, we combine BSBL with the spatial distribution properties of EEG to propose an RSBSBL. To ensure practicality, we design an automatic selection iteration strategy model to reduce the time cost caused by the inverse operation of large-size matrices.

For verification, RSBSBL was compared with some other methods with similar principles on the three BCI datasets. We used BLDA as a unified classifier for a fair comparison. The effectiveness of the proposed method was verified by the effectiveness of channel subsets and the character recognition performance.

We organize the rest of the paper as follows. Section “Materials and Methods” describes the principle and calculation process of the proposed algorithm. Section “Materials and Experiments” describes the dataset used and the data processing framework. Section “Results” shows the experimental results. Section “Discussion” further discusses the effectiveness of the selected channel subsets, character recognition performance, effectiveness of regional smoothing, time cost, and future work. Finally, section “Conclusion” gives the conclusion.

## Methods

Here, we show the principle and solution process of RSBSBL and give its flow of selecting channels. The input features of one channel are regarded as a block. Based on the BSBL, we considered the spatial distribution of EEG and divided different regions according to the location of the electrodes. The automatic selection mode of the iterative strategy is used to ensure practicality.

### Regional Smoothing Sparse Bayesian Learning

The EEG signals collected by the device are generally two-dimensional data after pre-processing. *N_c_* is denoted as the number of channels and *N_t_* as temporal points. Input data **X** contains *N* samples **x**_1_,**x**_2_,**x**_3_ …… **x**_*N*_ ∈ *R^D^*, where *D* = *N*_*t*_*N*_*c*_ represents the number of features in each sample. Then, **X** = [**x**_1_,**x**_2_,**x**_3_, … **x**_*N*_]^T^ ∈ *R*^*N* × *D*^ and **y** = [*y*_1_,*y*_2_,*y*_3_, …, *y*_*N*_]^T^ ∈ *R^N^* represent the corresponding labels, where *y*_*i*_ ∈ {1,−1} is the class label. Its mathematical model can be expressed linearly as follows:


(1) y=Xw+ε

where **w** = [*w*_1_,*w*_2_,*w*_3_ …… *w*_*D*_]^T^ is a learnable weight vector, ε is noise, and **X** can be replaced by Φ(**X**) expressed in the form of a kernel function. Assume ε ∼ 𝒩 (0, σ^2^**I**_*N*_), then **y** ∼ 𝒩 (**X**w, σ^2^**I**_*N*_) and its probabilistic framework is.


(2) $$\begin{array}{c} p\left(\text{y}|w,\sigma^{2}\right)={\left(2\pi \sigma^{2}\right)}^{-\frac{N}{2}}exp\left(-\frac{1}{2\sigma^{2}}{||\text{y}-\text{X}w||}_{2}^{2}\right) \end{array}$$

The RSBSBL adds the symmetric positive definite matrix in the variance term of the distribution that **w** obeys. The input data of one channel are regarded as a block. So, for the mathematical model (1), assume that **w**_*b*_(∀*b*) is mutually independent and Gaussian distributed.


(3) $$\begin{array}{c} p\left({\text{w}}_{b}|\gamma_{b},{\text{B}}_{b},\forall b\right)∼𝒩\left(0,\gamma_{b}{\text{B}}_{b}\right),b\in 1,\ldots ,N_{b} \end{array}$$

where **w**_*b*_ containing several *w_i_* is *b*th block of **w**, γ_*b*_ is a non-negative scalar that controls the variance of **w**_*b*_, **B**_*b*_ is a positive definite matrix reflecting the intra-block correlation, and *N_b_* is the number of blocks. Since the features of a channel are considered to be a block, *N*_*b*_ = *N*_*c*_.

In our case of EEG signal, *b* is the index of channels. In a channel of EEG signal with corresponding weight **w**_*b*_, it is assumed that all its feature weights share the same γ_*b*_ to control the variance of their distribution, and **B**_*b*_ controls the intra-block correlation.

In this case, we express the prior of **w** as *p*(**w**|γ, **B**) ∼ 𝒩 (0, **Σ**_0_), where **Σ**_0_ is


(4) $$\Sigma_{0}=\left[\begin{array}{ccccc} \gamma_{1}B_{1} & & & & \\ & . & & & \\ & & . & & \\ & & & . & \\ & & & & \gamma_{N_{b}}B_{N_{b}} \end{array}\right]$$

the posterior probability has been calculated by the Bayesian rule,


(5) $$\begin{array}{c} p\left(\text{w}|y,\sigma^{2},\gamma ,B\right)=\frac{p\left(\text{y}|w,\sigma^{2}\right)p\left(\text{w}|\gamma ,B\right)}{p\left(\text{y}|\sigma^{2},\gamma ,B\right)} \end{array}$$

and the corresponding variance and mean of the posterior probability density *p*(**w**|**y**, σ^2^, γ, **B**) ∼ 𝒩 (μ_**w**_, **Σ_w_**) can be described as


(6) $$\begin{array}{c} \Sigma_{w}={\left(\sigma^{-2}{\text{X}}^{\text{T}}\text{X}+\Sigma_{0}^{-1}\right)}^{-1} \end{array}$$

(7) $$\begin{array}{c} \mu_{w}=\sigma^{-2}\Sigma_{w}{\text{X}}^{\text{T}}\text{y} \end{array}$$

When *N* ≥ *D*, the Eqs (6) and (7) are suitable because the maximum size of the inverse matrix is D in this case. Now, we give the iterative ways when *N* < *D*. According to the matrix inversion formula and the matrix identity.


(8) $$\begin{array}{c} {\left(\text{E}+\text{FGH}\right)}^{-1}={\text{E}}^{-1}-{\text{E}}^{-1}\text{FG}{\left(\text{I}+{\text{HE}}^{-1}\text{FG}\right)}^{-1}{\text{HE}}^{-1} \end{array}$$

(9) $$\begin{array}{c} {\left(\text{I}+\text{EF}\right)}^{-1}\text{E}=\text{E}{\left(\text{I}+\text{FE}\right)}^{-1} \end{array}$$

we replace the Eqs (6) and (7) with the following equations:


(10) $$\begin{array}{c} \Sigma_{w}=\Sigma_{0}-\Sigma_{0}{\text{X}}^{\text{T}}{\left(\sigma^{2}\text{I}+\text{X}\Sigma_{0}{\text{X}}^{\text{T}}\right)}^{-1}\text{X}\Sigma_{0} \end{array}$$

(11) $$\begin{array}{c} \mu_{w}=\Sigma_{0}{\text{X}}^{\text{T}}{\left(\sigma^{2}\text{I}+\text{X}\Sigma_{0}{\text{X}}^{\text{T}}\right)}^{-1}\text{y} \end{array}$$

To find the iterative equation of the parameters **Θ** = {γ, **B**, σ^2^}, the expectation–maximization (EM) is used to maximize log *p* (**y**|**Θ**). The *Q* function is.


$$Q\left(\Theta \right)=E_{w|y,\Theta_{\text{old}}}\left[\text{log}p\left(y,\text{w}|\Theta \right)\right]=E_{w|y,\Theta_{\text{old}}}\left[\log p\left(\text{y}|w,\sigma^{2}\right)\right]+{\text{E}}_{w|y,\Theta_{\text{old}}}\left[\text{log}p\left(\text{w}|\gamma ,B\right)\right]$$

The first term of the *Q* function is related to σ^2^ and the second term is related to γ and **B**. Then, we can get the parameters iteratively by maximizing the **Q** function.


(13) $$\begin{array}{c} \sigma^{2}=\frac{{||\text{y}-\text{X}\mu_{w}||}_{2}^{2}+\sigma_{\text{old}}^{2}\left[D-\text{Tr}\left(\Sigma_{w}\Sigma_{0}^{-1}\right)\right]}{N} \end{array}$$

(14) $$\begin{array}{c} \gamma_{b}=\frac{\text{Tr}\left[{\text{B}}_{b}^{-1}\left(\Sigma_{w}^{b}+\mu_{w}^{b}{\left(\mu_{w}^{b}\right)}^{\text{T}}\right)\right]}{d_{b}},\forall b \end{array}$$

(15) $$\begin{array}{c} {\text{B}}_{re}=\frac{1}{g_{re}}\sum_{b\in G_{re}}\frac{\Sigma_{w}^{b}+\mu_{w}^{b}{\left(\mu_{w}^{b}\right)}^{\text{T}}}{\gamma_{b}},\forall re \end{array}$$

where *old represents the hyperparameter in the previous iteration, and the superscript *b* of $\mu_{w}^{b}$ and $\Sigma_{w}^{b}$ indicates the *b*th block in μ_**w**_ and **Σ_w_** with the size of *d*_*b*_ × 1 and *d*_*b*_ × *d*_*b*_ (*d_b_* is the number of elements in w_b_).

**FIGURE 1:**
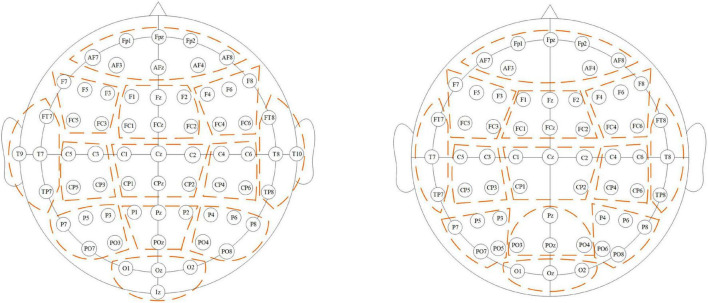
Region division. Channels belonging to a region are circled with the dotted line. The left subfigure shows the division for DS1 and DS2, while the right subfigure shows the division for DS3.

The potential similarity exists in the adjacent electrode signals considering the volume conduction effects in the brain (Hassan and Wendling, 2018). We assign the same **B**_*re*_ for channels with close locations for regional smoothing, and the region *G*_*re*_ contains *g*_*re*_ channels. As shown in Figure 1, all the channels are divided into 13 regions by position, and each region contains at least three channels. **B**_*re*_ is the average of blocks in region *re* (*re* ∈ [1, 13]).

We use a first-order Auto-Regressive (AR) process to model the intra-block correlation. Many applications have used the AR process to express it (Zhang and Rao, 2011; Zhang et al., 2013; Yin et al., 2020). Thus, to find a symmetric positive definite matrix to approximate **B**, it can be constrained to the following form of the Toeplitz matrix.


(16) $$\begin{array}{c} {\text{B}}_{re}≜\text{Toeplitz}\left(\left[1,r,\ldots ,r^{d_{b}-1}\right]\right)=\left[\begin{array}{ccccc} 1 & . & . & . & r^{d_{b}-1}\\ . & . & & & .\\ . & & . & & .\\ . & & & . & .\\ r^{d_{b}-1} & . & . & . & 1 \end{array}\right] \end{array}$$

Empirically calculate $r=\frac{m_{1}}{m_{0}}$, where *m_0_* is the average of the main diagonal of **B**_*re*_ and *m*_*1*_ is the average of the main sub-diagonal.

### Channel Selection Based on Regional Smoothing BSBL

Regarding the feature extracted from the same channel as a block, we perform RSBSBL to get the weight vector of features and design a channel selection based on the weight vector as Algorithm 1.

**Algorithm 1:** Regional Smoothing Sparse Bayesian Learning (RSBSBL).

**Input:** features **X**_*N* × (*N*_*c*_*N*_*t*_)_ and labels **Y**_*N* × 1_, where *N* denotes the number of samples, *N_c_* represents the number of channels, and *N_t_* is the number of features (sampling points) in one channel.
**Output:** sparse weights **w** and selected channels *C_s_*.
1: Choose an initial setting for σ^2^, γ, **B**. The block size is *N_t_*.
2: Set a shear threshold τ to obtain the sparsity weights.
3: **While** the convergence criterion is not satisfied, **do**
4: **If** *N* ≥ *N*_*c*_ × *N*_*t*_, **then**
5: Calculate **Σ_w_**, μ_**w**_, according to (6)(7).
6: **Else**
7: Calculate **Σ_w_**, μ_**w**_, according to (10)(11).
8: **End if**
9: Update σ^2^, γ, **B** according to (13)(14)(15)and (16).
10: **If** γ_*b*_ < τ, **then** γ_*b*_ = 0, γ_*b*_ ∈ γ.
11: $\sigma_{\text{old}}^{2}$ = σ^2^, **B**_old_ = **B**, γ_old_ = γ.
12: **End while**
13: *C_s_* = {*b*|γ_*b*_ > τ, *b* ∈ 1, 2, …, *N*_*c*_, γ_*b*_ ∈ γ}.
14: **Return** $\text{w}=\left\{\mu_{w}^{b}|b\in C_{s}\right\}$ and *C_s_*.


**FIGURE 2:**
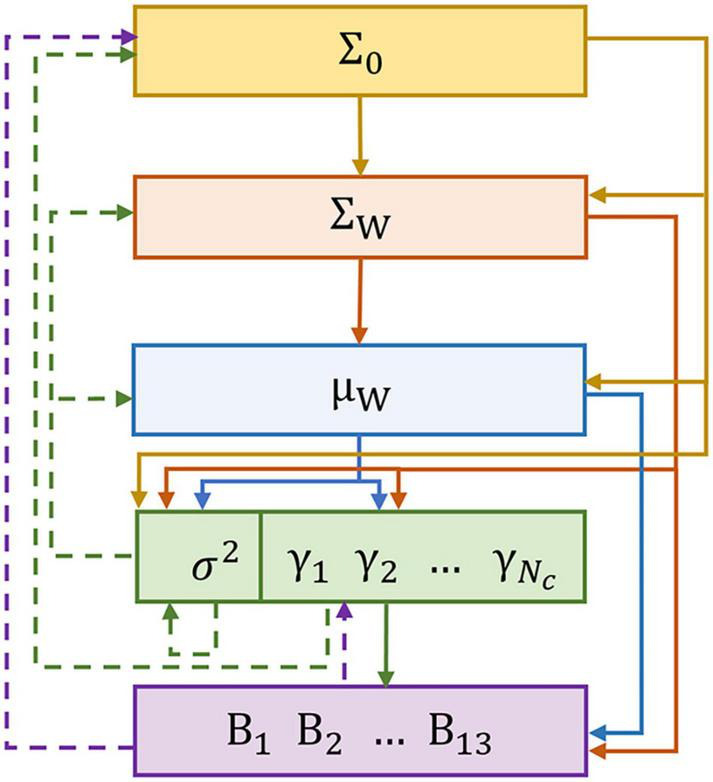
Parameter relationship graphical model in a single iteration. Parameters of the same color can be iterated simultaneously.

As shown in Algorithm 1, the parameters are initialized, and the shear threshold τ is set. Then, from Line 3 to Line 12, the algorithm iteratively solves BSBL and prunes the γ. Line 4 to Line 8 decide the calculation of **Σ_w_**, μ_**w**_, so that the large time cost caused by finding the inverse matrix of a large-size matrix can be alleviated. The parameters are updated on Line 9 and Line 10. Figure 2 illustrates the relationship between the parameters in a single iteration, where the parameters calculated simultaneously have the same color. The solid line indicates the passing relationship between the parameters of this iteration, and the dashed line indicates the passing relationship between the parameters of this iteration and the next iteration. After the parameters are calculated, in order to achieve the sparse block effect, make γ_*b*_ to *0* when γ_*b*_ is less than the threshold τ. Then, it comes into the next iteration until the convergence criterion is satisfied. Line 13 automatically selects the channels with γ_*b*_ greater than the shear threshold τ. Finally, the algorithm returns the selected channel and the corresponding weight vector.

The off-diagonal matrix **B** makes the weights **w** in the same block relevant in distribution. It means that the correlation of the features from the same channel can be reflected during the process. Moreover, the components of the temporal correlation of different channels in close locations are the same because the **B**_*re*_ of channels in the same region are shared. The sparsity of weights will form the units of channels. The features from one channel share the same weight distribution whose variance is controlled by γ. For practicality, up to five channels are removed in a single iteration when making a channel selection.

## Materials and Experiments

### Data Descriptions

Three datasets were used in this study to validate the proposed method. DS1 is BCI Competition II dataset IIb (one participant) (Blankertz et al., 2004) and DS2 is BCI Competition III dataset II (two participants) (Blankertz et al., 2006). DS3 is the EEG signal collected in our lab (12 participants). The stimulus numbers for each participant of the above three datasets are shown in Table 1.

**TABLE 1:** The stimulus numbers for each participant of DS1, DS2, and DS3.

Dataset	Stimulus category	Training dataset size	Test dataset size
DS1 P1.1	Target	1260	930
	Non-target	6300	4650
DS2 P2.1/P2.2	Target	2550	3000
	Non-target	12750	15000
DS3 P3.1-P3.12	Target	144	144
	Non-target	720	720

*Pi.j represents the jth participant in the ith dataset.*

**FIGURE 3:**
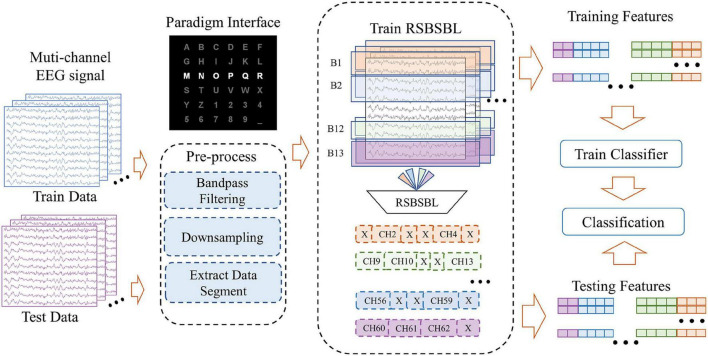
Diagram of the data processing framework, including pre-processing, channel selection, and classification. Using the block sparsity property of RSBSBL, we do pruning on the eligible channels by fitting the training data and labels.

DS1 and DS2 provided by the BCI Competition are public datasets and follow the same experimental paradigm of Farwell and Donchin, as shown in Figure 3. In a six-by-six character matrix containing 26 characters and 10 numbers, participants were asked to focus on a specified character in each trial (a trial is a set of stimuli that can support the output of a recognized character). They could do this by mentally counting the target stimuli’ number of flashes (intensifications). The paradigm continuously intensified and randomly scanned all rows and columns of the matrix at a rate of 5.7 Hz. Each row and column in the matrix was randomly intensified for 100 ms and was left blank for 75 ms. DS1 contained 42 training characters and 31 testing characters. The training set of DS2 contained 85 characters, and the testing set contained 100 characters. A trial for each character had 15 epochs to apply reliable spelling, and each epoch was comprised of 12 intensifications. Both datasets were collected using a 64-channel cap, filtered by 0.1–60 Hz, and digitized at a sampling rate of 240 Hz. DS1 and DS2 can be downloaded from the websites: http://www.bbci.de/competition/ii/ and http://www.bbci.de/competition/iii/.

DS3 was collected in our lab. Its paradigm was similar to the BCI Competition. It contained 26 characters and 10 numbers. DS3 consisted of 12 participants who were graduate students between the ages of 20 and 26 years, with normal or corrected-to-normal vision. The experiments used a 64-channel wireless EEG acquisition system (Neuracle, NeuSen W series, 59 EEG, 4EOG, 1ECG) to acquire data at the sampling rate of 1,000 Hz. In the paradigm, each row and column in the six-by-six matrix was randomly intensified for 80 ms and kept extinguished for 80 ms. A trial for each target character included four epochs, and each epoch had 12 intensifications. Participants were required to spell 36 characters. We randomly selected 18 characters as the training dataset and the rest as the test dataset.

### The Framework of Data Processing

Considering that some channels contain less task-relevant information but more noise, it is vital to use a reasonable method to select the most effective channels. This study compares the proposed RSBSBL with two empirical channel sets (Set 1 and Set 2) (Krusienski et al., 2008), LASSO, GLASSO, and SBL in the case of using the same pre-processing process and classifier. Set 1 includes Fz, Cz, Pz, Oz, PO7, and PO8. Set 2 includes Fz, FCz, Cz, C3, C4, CPz, Pz, P3, P4, P7, P8, POz, PO3, PO4, PO7, PO8, Oz, O1, and O2.

Figure 3 shows the diagram of the data processing framework, which includes three main parts: (1) pre-processing, (2) channel selection, and (3) classification. DS1 and DS2 shared the same pre-processing: bandpass filtering of data from 0.5 to 20 Hz and downsampling by a factor of 5. Then, the sampling rate of the data was 48 Hz. We intercepted 0–667 ms after each stimulus as the primary analysis objective was to obtain 32 sampling points for each stimulus. For the DS3, the 59-channel dataset that went through 0.5–20 Hz bandpass filtering was down-sampled to 50 Hz and the data segment from 0 to 600 ms was taken after stimulation to obtain 30 sampling points for each stimulus. Thus, denoting the number of channels as *N_c_* and number of signal sampling points as *N_t_*, a 1 × *D* feature matrix was obtained for each stimulus, where *D* = *N*_*t*_*N*_*c*_. A feature matrix was labeled “1” only if the corresponding stimulus belongs to the row or column of the target characters. Otherwise, the label was assigned to “0.”

The typical classification methods of P300 include traditional machine learning methods and neural network-based methods. Traditional machine learning can achieve outstanding performance with less complexity. This study regarded BLDA as a unified classifier for different channel selection algorithms.

### Parameter Setting

The optimal combination of parameters was determined by a 10-fold cross-validation. There were two modes of the selected channel number in the experiment for the channel selections: automatic and fixed. When the channel number was determined automatically, we used a threshold to determine the channel number. For LASSO and SBL, the absolute values of the feature weights in one channel were summed up to represent the importance of the channel. The threshold equaled the mean minus 0.5 times the standard deviation of the channel importance values, and the channels with importance values higher than the threshold were selected. As for GLASSO and RSBSBL, automatic channel selection had been enabled in the methods. When the number of selected channels is fixed (*M* channels were selected), we used the same way to evaluate each channel. For all the four methods, the absolute values of the feature weights **w** of each channel were summed, and the top *M* channels were selected in descending order.

### Evaluation

We used character recognition accuracy to evaluate the performance of a classification. The character recognition accuracy is defined as follows:


(17) $$\begin{array}{c} Acc=\frac{C_{test\_correct}}{C_{test\_total}} \end{array}$$

where *C*_*test_total*_ represents the total number of characters in the test dataset, and *C*_*test_correct*_ is the sum of all the correctly predicted characters. Besides, to evaluate the significance of performance difference, we introduced a non-parametric statistical hypothesis test, the Wilcoxon signed-rank test. The Wilcoxon signed-rank test can be used as an alternative to the paired *t*-test for matched pairs when the population cannot be assumed to be normally distributed. The significance of the pairs can be confirmed when the corresponding *p*-value is less than 0.05.

## Results

We evaluated the performance of the proposed method on the three datasets. The results covered the experiments of automatic channel selection and the experiments of selecting *M* channels. For further analysis, we also evaluated the sensitivity of the parameters of the proposed method.

### Results of Automatic Channel Selection

Channel selection is supposed to reserve channels with more helpful information and exclude the channels with more noise. According to the data processing, we chose a unified classifier to verify the performance of different methods for a fair comparison. In Table 2, we compared the character recognition accuracy of each method on the three datasets, and the number of selected channels was automatically determined as described in section “Parameter Setting.” Set 1 and Set 2 are empirical subsets of channels (Set 1 contains 6 channels and Set 2 contains 19 channels). The best results were marked in bold, and the number of channels selected for each participant is presented in the corresponding parentheses.

**TABLE 2:** Character recognition accuracy (%) (number of channels) and Wilcoxon signed-rank test comparisons for DS1, DS2, and DS3 when each compared method was used for channel selection.

Participant	Methods					
	Set 1	Set 2	LASSO	GLASSO	SBL	RSBSBL
P1.1	**100.00**	**100.00**	**100.00** (43)	**100.00** (54)	**100.00** (36)	**100.00** (29)
P2.1	80.00	92.00	96.00 (43)	98.00 (64)	97.00 (44)	**99.00** (44)
P2.2	90.00	92.00	93.00 (41)	95.00 (56)	93.00 (39)	**96.00** (45)
Average	85.00	92.00	94.50 (42.00)	96.50 (60.00)	95.00 (41.50)	**97.50** (44.50)
P3.1	55.56	61.11	83.33 (39)	**88.89** (22)	83.33 (40)	**88.89** (15)
P3.2	50.00	61.11	77.78 (39)	72.22 (37)	66.67 (42)	**94.44** (14)
P3.3	72.22	72.22	72.22 (42)	72.22 (38)	72.22 (39)	**94.44** (13)
P3.4	72.22	77.78	77.78 (35)	**83.33 (24)**	77.78 (39)	77.78 (18)
P3.5	55.56	61.11	83.33 (42)	**88.89 (23)**	77.78 (40)	**88.89** (14)
P3.6	44.44	44.44	72.22 (39)	72.22 (24)	83.33 (40)	**88.89** (40)
P3.7	66.67	77.78	**83.33** (41)	**83.33** (31)	72.22 (38)	**83.33** (15)
P3.8	72.22	77.78	72.22 (40)	77.78 (16)	77.78 (40)	**88.89** (13)
P3.9	61.11	66.67	77.78 (42)	83.33 (23)	77.78 (38)	**88.89** (15)
P3.10	72.22	**94.44**	88.89 (42)	88.89 (32)	88.89 (41)	**94.44** (15)
P3.11	61.11	66.67	50.00 (36)	72.22 (20)	50.00 (40)	**77.78** (19)
P3.12	38.89	83.33	83.33 (40)	83.33 (24)	88.89 (39)	**94.44** (13)
Average	60.19	70.37	76.85 (39.75)	80.55 (26.17)	76.39 (39.67)	**88.43** (17.00)
*p*-value	**0.002**	**0.005**	**0.005**	**0.013**	**0.003**	–

*Pi.j represents the jth participant in the ith dataset. The number of selected channels is in parentheses. The highest classification accuracy of each participant of different methods is indicated in bold. p-value is the results of Wilcoxon signed-rank test. Set 1 includes F_z_, Cz, Pz, Oz, PO7, and PO8. Set 2 includes Fz, FCz, Cz, C3, C4, CPz, Pz, P3, P4, P7, P8, POz, PO3, PO4, PO7, PO8, Oz, O1, and O2.*

For DS1, RSBSBL selected the minimum number of channels when the classification accuracy of all the methods was 100%. For DS2, RSBSBL had the highest average accuracy, 97.50%, which was 1.00% higher than the second-ranked GLASSO. Although SBL selected fewer channels than others, the average recognition accuracy was 95.00%.

For DS3, RSBSBL as a channel selection method could bring higher accuracy with BLDA in 11 participants among 12 and got 88.43% average accuracy by eliminating insufficient data than using all channels. It outperformed the second-ranked GSBL on an average by 7.88% and selected the fewest channels as 17. We evaluated the significance of the classification performance of DS3 *via* the Wilcoxon signed-rank test and found that the proposed method performed significantly better than others (RSBSBL vs. LASSO: *p* = 0.005 < 0.05; RSBSBL vs. GLASSO: *p* = 0.013 < 0.05; RSBSBL vs. SBL: *p* = 0.003 < 0.05).

### Results of Selecting M Channels

To further compare the effectiveness of the four methods, we compared the recognition results of the algorithms when *M* channels were selected (*M* = [4, 8, 12, 16]). Top *M* channels were selected by ranking the corresponding channels according to the sum of the absolute values of the feature weights. The classifiers were retrained with the data with the selected channel. It was supposed that the number of channels *M*′ automatically selected by the method was less than the value of *M*. In that case, the latest deleted M-M′ channels are added according to the order in which they were deleted during the iteration of the method.

**FIGURE 4:**
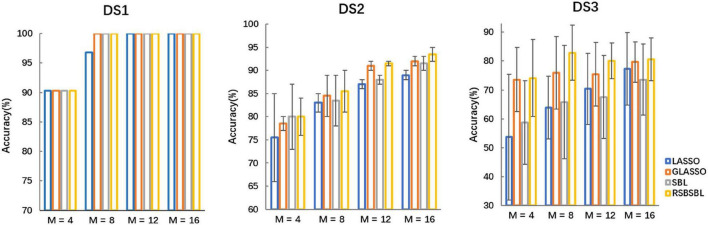
The average recognition accuracy of the four methods on DS1, DS2, and DS3 when *M* channels are selected, where *M* = [4, 8, 12, 16]. The error bars are the standard deviations for DS2 and DS3.

Figure 4 shows the accuracy of each method on DS1, DS2, and DS3, with the horizontal coordinates of the bars indicating the selection of the top *M* channels. For DS1, the accuracy of all the methods was the same except that the accuracy of SBL was 96.77% when eight channels were selected, and it was lower than others. For DS2, SBL and RSBSBL obtained better performance with 80% average recognition accuracy when four channels were selected. When 8, 12, and 16 channels were selected, GLASSO obtained an average recognition accuracy of 78.5, 84.5, 91, and 92%, respectively, and RSBSBL obtained a better performance of 80, 85.5, 91.5, and 93.5%, respectively. For DS3, GLASSO obtained average recognition accuracy of 73.61, 75.93, 75.46, and 79.63% when 4, 8, 12, and 16 channels were selected, respectively. Moreover, RSBSBL obtained the best performance of 74.07, 82.87, 80.09, and 80.56%, respectively. The average recognition accuracies of LASSO, GLASSO, and RSBSBL on DS3 with *M* = 16 were 77.31, 79.63, and 80.56%, respectively. The results of experiments with the fixed number of selected channels revealed that the feature weights generated by RSBSBL could provide more reasonable guidelines for the channel selection.

**FIGURE 5:**
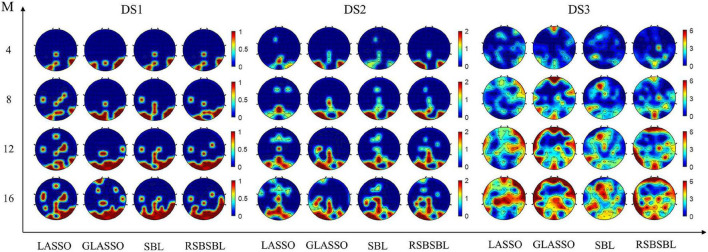
The scalp distribution of the four methods on DS1, DS2, and DS3 when *M* channels are selected. The contribution value of each channel is equal to the sum of the selected numbers among all participants in the dataset. The color changes from red to blue, indicating that the channel is selected less often.

We counted the selected channels at the same location and used it to describe the number of times a channel has been selected in the dataset. If 6 of the 12 participants’ selected channels contain Pz, then the contribution value of the channel corresponding to the Pz electrode is 6. Figure 5 indicates the scalp distributions of the contribution value of channels on DS1, DS2, and DS3. The color changes from red to blue, indicating that the channel was selected less often. As shown in Figure 5, when the number of selected channels was small (*M* = 4, 8), RSBSBL selected the occipital and parietal electrodes more often. It shows that, in addition to the P300 potential, the early visual components also contribute to a classification in the paradigm (Blankertz et al., 2011).

### Parameter Sensitivity

**FIGURE 6:**
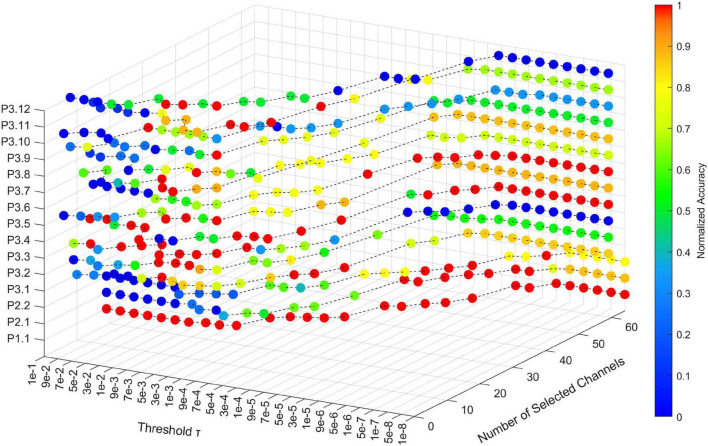
The effect of shear threshold τ in RSBSBL on the number of selected channels and accuracy. The *x*-axis indicates the number of selected channels, the *y*-axis indicates the value of τ, and the *z*-axis indicates the participant ID. The color of the sphere represents the normalized recognition accuracy for each participant with different thresholds.

In RSBSBL, γ_*b*_ smaller than the threshold τ was set to zero, indicating that τ determines the pruning strength. We analyzed the change in the number of channels selected and the recognition results when τ is assigned different values in the range 10^−8^ to 10^−1^. The recognition accuracy of each participant varying with τ was normalized to highlight the location of the optimal threshold. Figure 6 illustrates the effect of the threshold on the proposed method. The *x*-axis indicates the number of selected channels, the *y*-axis indicates the value of τ, and the *z*-axis indicates the participant ID. The color changes from red to blue, indicating that the point corresponds to a higher to lower normalized accuracy.

As shown in Figure 6, the number of channels selected by each participant increased as the threshold value decreased. When the threshold was less than or equal to 10^−6^, the number of selected channels was the original number in the dataset, and the algorithm loses the ability to select the channels automatically. Therefore, 10-fold cross-validation can be used to select the optimal parameter values in the range of 10^−6^ to 10^−1^. From the curves corresponding to P3.2, P3.3, P3.7, and P3.12, using selected channels can obtain better recognition accuracy than using all the channels, which proves that channel selection can remove weak task-relevant and noisy channels to improve the classification accuracy.

## Discussion

The experimental results on the three datasets illustrated that the proposed RSBSBL as a channel selection algorithm could automatically screen out effective channels and get the best overall performance among all the compared methods.

### Effectiveness of Channel Subsets

Fabiani et al. (1987) confirmed that the visual P300 paradigm should at least include Fz, Cz, Pz electrodes signed as the 10–20 international electrode system. Krusienski et al. (2008) and McCann et al. (2015) made sure that Fz, Cz, Pz, Oz, PO7, and PO8 corresponded to the parietal and occipital regions of the brain that take a significant part in the recognition of P300 signals. In Table 2, Set 1 and Set 2 represent two empirical channel subsets. Set 1 includes Fz, Cz, Pz, Oz, PO7, and PO8. Set 2 includes Fz, FCz, Cz, C3, C4, CPz, Pz, P3, P4, P7, P8, POz, PO3, PO4, PO7, PO8, Oz, O1, and O2. It can be seen that for many participants (P2.1, P2.2, P3.1, P3.2, P3.5, P3.6), the character recognition accuracy was lower when the empirical channel subsets were used. The empirical selection may not include some channels that contribute to the classification. The channels assumed to reflect visual components and also some frontal channels contribute to the classification for some participants. It also indicates the lower robustness of the empirical channel subset. In Figure 5, the scalp mapped according to channel selection of RSBSBL could be observed with high values in Pz, P3, P4, O1, O2, Oz, PO7, PO8, and POz regions. These electrodes are very similar to the abovementioned electrodes, which are closely related to the visually induced ERPs. The P1, N1, and N2 components are mainly concentrated in the parietal and occipital regions. And the central distribution of P2 and P3 is elongated along the midline electrodes (Blankertz et al., 2011). It can be assumed that a multitude of ERP components is affected by attention to the target and utilized by classifiers rather than just the P300 (Treder and Blankertz, 2010). In addition, it can be found from Figures 5, 6 that many participants in DS3 had poorer classification using full-channel data compared to DS1 and DS2, and their topographic maps select more frontal channels when *M* = 8, 12,16. This phenomenon may be due to the effect of eye artifacts and noise during the experiment.

### Character Recognition Performance

Table 2 and Figure 3 show the superiority of RSBSBL in channel selection. When the number of channels was determined automatically, the proposed method achieved the highest average recognition accuracy of 100, 97.5, and 88.43% for DS1, DS2, and DS3, with the lowest average number of channels on DS1 and DS3. The RSBSBL achieved better performance than the compared methods when selecting the channels with the fixed number, and the average accuracies of 90.21, 80, and 74.07% were obtained with the top four selected channels on the three datasets.

To verify the performance of RSBSBL, we compared the proposed method with the state-of-the-art developments in recent years on DS2, as shown in Table 3. Most of them are based on evolutionary computational algorithms (Kee et al., 2015; Khairullah et al., 2020; Tang et al., 2020; Martinez-Cagigal et al., 2022). The channel selection methods and classifiers used in each study are shown in the table.

**TABLE 3:** Character recognition accuracy (%) of comparison with state-of-the-art results (DS2).

Author	Channel selection method	Classification method	Accuracy
Kee et al., 2015	NSGA-II	BLDA	94.9%
Khairullah et al., 2020	BPSO	Ensemble LDA	97.0%
Tang et al., 2020	RF-GA	CNN	96.9%
Martinez-Cagigal et al., 2022	BMOPSO	LDA	92.5%
	PEAIL	LDA	94.0%
Our method	RSBSBL	BLDA	97.5%

*NSGA-II, Non-dominated sorting genetic algorithm II; BPSO, binary particle swarm optimization; GA, genetic algorithm; BMOPSO, binary multi-objective particle swarm optimization; PEAIL, Pareto Evolutionary Algorithm based on Incremental Learning.*

The shear threshold τ significantly impacted the final results, so cross-validation was required to determine the optimal parameters. According to the analysis of parameter sensitivity, as shown in Figure 6, the recommended threshold selection range was [10^−6^, 10^−1^]. Besides, Figure 6 reflects the variation of character recognition accuracy with the shear threshold for each participant. Compared with others, P3.2, P3.3, P3.9, P3.10, and P3.12 cannot achieve the best recognition accuracies with the full channels, which implies that the EEG signals of these participants have more channels with noise, and these channels are not conducive to signal classification. As shown in Table 2, when determining the number of channels automatically, RSBSBL can achieve the best recognition accuracies of them with the corresponding number of selected channels of 14, 13, 15, 15, and 13, respectively. It confirms that RSBSBL can remove unfavorable channels and improve the recognition accuracies.

### Effectiveness of Regional Smoothing

To verify the effectiveness of regional smoothing, we conducted further controlled experiments on the three datasets, and the results are shown in Table 4. Case 1 represents that B is a unit matrix, implying that no temporal correlation is considered. Case 2 has the same B for all blocks, indicating that all channels share the same B. Case 3 has a different B matrix for each block, showing that regional smoothing is no longer done. The comparison between Case 3 and Case 1 in Table 4 illustrates the improvement of the model due to temporal correlation. The comparison between our algorithm and Cases 3 and 1 indicates the improvement brought by region smoothing. The“*” in Table 4 represents a significant difference in our method after Wilcoxon signed-rank test (RSBSBL vs. Case 1: *p* = 0.015 < 0.05; RSBSBL vs. Case 2: *p* = 0.031 < 0.05; RSBSBL vs. Case 3: *p* = 0.124).

**TABLE 4:** The average character recognition accuracy (%) (number of channels) comparisons on three datasets.

Method	DS1	DS2	DS3
Case 1	100.00 (30)	94.50 (35.50)	82.41 (17.75)*
Case 2	100.00 (27)	96.00 (36.50)	82.41 (18.67)*
Case 3	100.00 (27)	96.50 (47.50)	85.65 (16.33)
Our method	100.00 (29)	97.50 (44.50)	88.43 (17.00)

Case 1: B is the unitary matrix. Case 2: All blocks have the same B. Case 3: The B of each block is different. “*” represents a significant difference with our method after Wilcoxon signed-rank test (p < 0.05).

### Time Costs and Limitations

As described in sections “Data Descriptions” and “The Framework of Data Processing,” for DS1 and DS2, *N*_*t*_ = 32 and *N*_*c*_ = 64 after pre-processing, then we can get a 1 × *D* (*D* = *N*_*t*_*N*_*c*_ = 2048) vector for each stimulus. As shown in Table 1, in the training datasets of DS1 and DS2, the total number of stimuli was 7,560 and 15,300, which is larger than the number of features *D*. For DS3, *N*_*t*_ = 30 and *N*_*c*_ = 59 after pre-processing, then the feature is a 1 × *D* (*D* = *N*_*t*_*N*_*c*_ = 1770) vector. In Table 1, in the training datasets of DS3, the total number of stimuli was 864, which is smaller than the number of its features.

**FIGURE 7:**
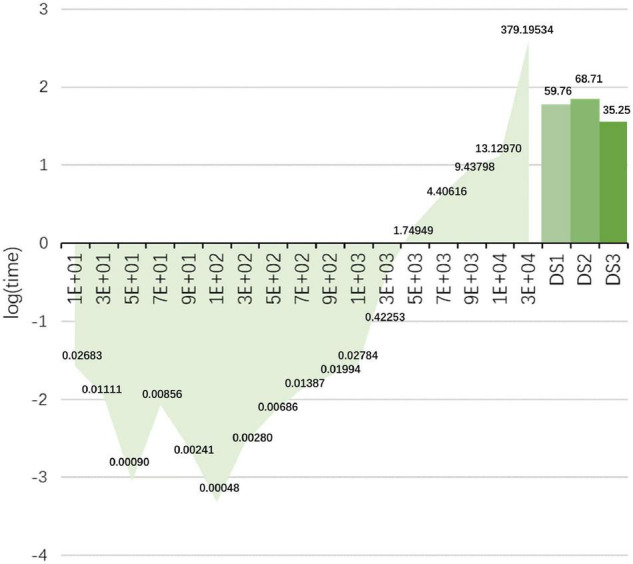
Changes in the run-time (s) of matrix inversion when the size of the matrix increases. In the left part, the horizontal axis represents the size of the square array. The vertical axis is the value after taking the logarithm of the time. The bar chart represents the average time cost of the proposed method on the three datasets.

In a preliminary study, we found that inappropriate iterations can make the algorithm to have a large time cost [e.g., using equations (10) and (11) on DS1 and DS2]. Therefore, a strategy of automatic selection of the iteration method is used to avoid this problem. In Figure 7, we analyze the variation of the matrix inversion run-time when the size of the matrix increases (the matrix is a square matrix). In the left part, the horizontal axis represents the size of the square matrix. The vertical axis is the value after taking the logarithm of the time, and the actual time (s) is also indicated in the figure. It can be noticed that the time spent on matrix inversion is more than 1 s when the matrix size is larger than 3,000 × 3,000. Therefore, we consider that the method may not be suitable for data with numbers of features and samples larger than 3,000. Of course, this problem can be solved by reducing the number of features and optimizing the iteration steps. The right bar in Figure 7 indicates the average time cost of the proposed method on the three data sets, which is acceptable.

### Future Work

The sparse Bayesian algorithm can make the sparsity of the algorithm change by changing the prior distribution of **w** (Tipping, 2001). Zhang et al. (2015) used the Laplace distribution instead of the traditional Gaussian distribution for the classification of P300 signals using SBL. Therefore, RSBSBL can change the prior of the weights to make the sparsity stronger in the future, such as the Gamma distribution. The proposed method used the EM algorithm for iteration, and there is still room for improvement in the computational speed. In the future, we will also explore the suitability of the proposed method for other ERPs.

## Conclusion

This study proposed a novel channel selection method, namely RSBSBL, which improved the original BSBL and obtained the assigned sparse weights. While considering the temporal correlation of sampling points of the same channel, it exploits the spatial distribution characteristics of the electrodes so that channels in adjacent regions share a positive definite matrix to get regional smoothing. Also, we discussed the efficiency of RSBSBL in the channel selection and design an automatic selection iteration strategy model to reduce the time cost caused by the inverse operation of the large-size matrix. The experimental results on three datasets indicate that RSBSBL can select appropriate channels, leading to high recognition accuracy. We will conduct future studies to improve the robustness of this algorithm.

## Data Availability Statement

The original contributions presented in the study are included in the article/supplementary material, further inquiries can be directed to the corresponding author.

## Ethics Statement

The studies involving human participants were reviewed and approved by the Ethics Committee of East China University of Science and Technology. The patients/participants provided their written informed consent to participate in this study. Written informed consent was obtained from the individual(s) for the publication of any potentially identifiable images or data included in this article.

## Author Contributions

XZ was the main author to raise the idea of the manuscript, designed the experimental procedure, and collected the original dataset. JJ made effective suggestions on the manuscript’s structure and provided the experimental site. RX has embellished the language of the manuscript and made key suggestions. SL and HS were involved in revising the manuscript’s results section. XW and AC provided inputs for optimizing the data processing flow. All authors contributed to the manuscript revision, and read and approved the submitted version.

## Conflict of Interest

RX is employed by the company g.tec medical engineering GmbH. The remaining authors declare that the research was conducted in the absence of any commercial or financial relationships that could be construed as a potential conflict of interest.

## Publisher’s Note

All claims expressed in this article are solely those of the authors and do not necessarily represent those of their affiliated organizations, or those of the publisher, the editors and the reviewers. Any product that may be evaluated in this article, or claim that may be made by its manufacturer, is not guaranteed or endorsed by the publisher.
